# Strength of Evidence to Support Decision-Making on the Use of Digital Mental Health Technologies in NICE Evaluations: Cross-Sectional Analysis of Studies

**DOI:** 10.2196/85635

**Published:** 2026-04-07

**Authors:** Gareth Hopkin, Holly Coole, Francesca Edelmann, John Powell, Mark Salmon, Sophie Cooper

**Affiliations:** 1 Science Evidence and Analytics Directorate, National Institute for Health and Care Excellence, 3 Piccadilly Place, Manchester, M1 3BN, United Kingdom, 44 7973 970 534; 2 Software Team, Healthcare Quality and Access Group, Medicines and Healthcare products Regulatory Agency, London, United Kingdom; 3 Nuffield Department of Primary Care Health Sciences, University of Oxford, Oxford, United Kingdom

**Keywords:** digital technologies, mental health, evidence gaps, health technology assessment, outcomes research

## Abstract

**Background:**

Digital mental health technologies (DMHTs) are playing an increasing role in mental health services. The quality of evidence for DMHTs is variable, and there are concerns that evidence is not sufficient to support decision-making.

**Objective:**

This study used a cross-sectional analysis of evidence supporting DMHTs included in National Institute for Health and Care Excellence (NICE) evaluations to examine the strength of evidence available for decision-making.

**Methods:**

We identified all NICE evaluations relating to DMHTs by reviewing details of published NICE evaluations on the NICE website. From each of these evaluations, we identified included DMHTs and reviewed committee documentation to identify studies that provided supporting evidence for each of these technologies. We extracted information on a series of items relating to study quality and summarized the characteristics of evidence both at the level of individual studies and across the package of evidence from multiple studies supporting DMHTs. We also identified key evidence gaps in available evidence.

**Results:**

We included nine NICE evaluations relating to anxiety, depression, psychosis, insomnia, attention deficit hyperactivity disorder (ADHD), and tic disorders. These evaluations included 30 DMHTs and referenced 78 supporting studies. We identified common evidence gaps relating to effectiveness compared to relevant comparators, use of appropriate outcomes, including health-related quality of life, cost of delivery, and impact on resource use, and reporting of adverse events.

**Conclusions:**

Our study highlights that some DMHTs have been supported by high-quality studies and that evidence to support DMHTs is likely to be developed across a series of studies. However, there are often key evidence gaps that need to be addressed to provide a stronger case for adoption. Developers should ensure that they consider these gaps while planning evidence generation, and where possible, address them earlier in the product lifecycle.

## Introduction

Digital mental health technologies (DMHTs) are playing an increasing role in mental health services and are seen as a way to respond to increasing pressure on services by expanding access to care and improving outcomes for patients [1,2]. They are available for a range of mental health conditions, from more common mild-to-moderate conditions to severe and enduring presentations, and can play a role in triaging and diagnosing, providing treatment, and helping people to track and manage their mental health over time [3].

For DMHTs to be adopted in health services, they should be supported by evidence to demonstrate that they improve outcomes and deliver value for money [4]. However, there are concerns that evidence supporting DMHTs is often limited. A comprehensive review of digital health interventions across a range of health conditions identified evidence gaps relating to longer-term health outcomes, quality of life and wellbeing, and cost of delivery and downstream resource use [5]. A lack of high-quality study designs and research in these areas has also been highlighted for DMHTs [6,7]. There are also other challenges for evidence generation for DMHTs due to complexities associated with mental health research [8,9]. These include selection of appropriate outcome measures [9], inadequate monitoring and reporting of adverse events [10], and ensuring user engagement with high rates of early discontinuation of DMHTs [11].

Previous research in this area has mainly focused on the design and quality of individual studies with a focus either on particular types of studies (eg, randomized controlled trials), types of interventions (internet and app-based interventions), or populations (eg, psychosis). However, this approach may not reflect the strength of evidence supporting DMHTs. This is because it is unlikely to reflect the overall package of evidence for DMHTs, which can be generated across multiple studies addressing different issues relating to safety, effectiveness, and cost-effectiveness. These packages of evidence are of particular importance as they are used as the basis for decisions about the use of DMHTs in health services.

In many countries, decisions about the use of DMHTs in health services are guided by recommendations from health technology assessment (HTA) agencies [8]. These agencies follow structured approaches to identifying, synthesizing, and appraising evidence that is relevant to the use of DMHTs in specific contexts in order to develop recommendations. Documentation generated in the process of these evaluations can provide valuable information on the strength of evidence supporting DMHTs and areas where there are often evidence gaps. The National Institute for Health and Care Excellence (NICE) is England’s HTA agency and provides guidance on the use of health technologies, including digital health technologies, in the National Health Service (NHS) in England [12,13]. Topics for evaluation are identified through engagement with stakeholders from across the health and care system and prioritized based on national priorities, the potential value of NICE guidance, and the availability of evidence to support an evaluation. NICE evaluations are supported by evidence summaries that provide information on studies that support included technologies [14]. These studies are identified using systematic review methods and additional information provided by developers. This information is publicly available on the NICE website for each evaluation, along with other information that informs committee decision-making.

For digital health technologies, NICE employs a life-cycle evaluation approach, and the type of assessment reflects the stage that a technology has reached in its lifecycle [14]. For DMHTs, the stages of the lifecycle that are currently most relevant are early use and routine use. For early use, technologies can be recommended for use while further evidence is generated. These recommendations are accompanied by an Evidence Generation Plan that outlines key evidence gaps and approaches that could be used to address these gaps. Technologies that have a more mature evidence base can be recommended for routine use. Technologies can also be recommended for use in research only.

### Objective

In this paper, we completed a cross-sectional analysis of evidence supporting DMHTs included in NICE evaluations to examine the strength of evidence to support decision-making both at the level of individual studies and across the package of evidence from multiple studies supporting DMHTs. We also aimed to identify common evidence gaps that, if addressed, could provide more certainty on the impact of using DMHTs.

## Methods

### Ethical Considerations

This study did not involve human participants and therefore, ethics approval and informed consent were not required.

### Study Design and Information Sources

We used a cross-sectional analysis based on NICE evaluations for DMHTs. We identified all NICE evaluations relating to DMHTs published before September 2025. These were identified based on internal knowledge and a review of published guidance on the NICE website [15]. We defined digital mental health as any topic that related to the use of software to support triage, diagnosis, treatment, or ongoing management of mental health, behavioral, and neurodevelopmental conditions. We excluded topics related to the management of physical health conditions, even if these related to mental health populations.

From each NICE evaluation, we identified included DMHTs and reviewed committee documentation to identify studies that provided supporting evidence for each of these technologies. As this review is focused on studies relating to effectiveness and cost-effectiveness, we excluded studies if they reported only patient engagement or experience and did not report outcomes relating to clinical symptoms, health-related quality of life (HRQoL), functioning, well-being, or cost and resource use. For diagnostic technologies, we also excluded studies that provided information only on diagnostic accuracy due to differences in assessing quality for these study designs, including the need to consider appropriate reference standards, measures of accuracy, and performance at threshold settings.

### Data Extraction

For each identified study, we extracted information on a series of items relating to study quality. The items were selected because they provide a broad overview of the quality of research methodology and reporting and are commonly included across risk of bias tools and preferred reporting items checklists [16,17]. These items have also been included in similar reviews of evidence supporting regulatory approval [18].

Data on these items were extracted from publicly available supporting documentation on the NICE website (eg, committee papers, assessment reports) and from published versions of supporting studies. Where information was contained in multiple sources, we relied primarily on the original publication to ensure accuracy. We also recorded whether committee documentation mentioned ongoing studies that could provide additional evidence for a technology after the conclusion of the evaluation. For evaluations for early use, we also extracted information on additional evidence that was essential or could support further review from Evidence Generation Plans.

### Data Synthesis

We report the number of identified NICE evaluations for DMHTs, the number of DMHTs included within these evaluations, and the total number of included studies. For each item relating to study quality, we categorized each study according to relevant characteristics and report the number and proportion of studies with these relevant characteristics. We also reported the number of studies supporting each identified DMHT and the number and proportion of DMHTs that were supported by evidence indicating higher quality or relevance to decision-making across a subset of items. These items were based on previous research, which has examined the quality of studies supporting regulatory submission [18] and align with consensus-based reporting guidelines [16]. Finally, we report evidence gaps identified in NICE evaluations where DMHTs were for early use and had recommendations for use with further evidence generation.

## Results

### Overview of NICE Evaluations

We reviewed nine NICE evaluations on DMHTs (Table 1). These covered a broad range of conditions, including anxiety, depression, psychosis, insomnia, attention deficit hyperactivity disorder (ADHD), and tic disorders.. They also covered a range of uses, including triage, diagnosis, treatment, and ongoing management and support. Recommendations were available on 30 DMHTs. Four of these technologies were included in multiple evaluations for different populations. Two recommendations were made for routine use, 20 recommendations were made for use while further evidence is generated, and 20 recommendations were made for use in research only. Recommendations on two technologies have been withdrawn since initial publication, as the technologies are no longer available in England.

**Table 1.** Included NICE^a^ evaluations.

Reference	Evaluation	Published
HTE3 [19]	Guided self-help digital cognitive behavioural therapy for children and young people with mild to moderate symptoms of anxiety or low mood	February 2023
HTE8 [20]	Digitally enabled therapies for adults with depression	May 2023
HTE9 [21]	Digitally enabled therapies for adults with anxiety disorders	May 2023
HTE15 [22]	Virtual reality technologies for treating agoraphobia or agoraphobic avoidance	November 2023
HTE17 [23]	Digital health technologies to help manage symptoms of psychosis and prevent relapse in adults and young people	March 2024
HTE25 [24]	Digital therapy for chronic tic disorders and Tourette syndrome	May 2025
HTE30 [25]	Digital front door technologies to gather information for assessments for NHS Talking Therapies for anxiety and depression	July 2025
DG60 [26]	Digital technologies for assessing attention deficit hyperactivity disorder (ADHD)	October 2024
MTG70 [27]	Sleepio to treat insomnia and insomnia symptoms	May 2022

a NICE: National Institute for Health and Care Excellence.

### Characteristics of Identified Studies

We identified a total of 78 studies related to 25 DMHTs. Seven of these studies provided support for technologies across two evaluations. Of the studies, 72 were publicly available. Six studies were not publicly available but were provided in confidence during NICE evaluations, and some information on these was reported in publicly available supporting documentation. Two technologies were also supported by confidential responses to requests for information during the NICE evaluation. These responses may include additional evidence that is not publicly available and was not available to us, so could not be included in this review. Summary characteristics of included studies are described in Table 2, Table 3, and Table 4.

**Table 2.** Characteristics of studies relating to study design.

Study Characteristics	Studies, n (%)
Publicly available	
Yes	72 (92.3)
No	6 (7.7)
Reporting of conflicts	
Reported	68 (87.2)
Not reported	4 (5.1)
Not available	6 (7.7)
Funding sources	
Industry	20 (25.6)
Insurer	1 (1.3)
Public	38 (48.7)
Third sector	11 (14.1)
Not available	11 (14.1)
Trial registration	
Registered	37 (47.4)
Not registered	35 (44.9)
Not available	6 (7.7)
Recruitment setting	
England	43 (55.1)
Rest of UK	9 (11.5)
International	27 (34.6)
Not available	5 (6.4)
Centers	
Single center	20 (25.6)
Multi-center	52 (66.7)
Not available	6 (7.7)
Design	
Randomized controlled	42 (53.8)
Individual	41 (52.6)
Cluster	1 (1.3)
Non-randomized controlled	11 (14.1)
Cohort	10 (12.8)
Individual	1 (1.3)
Non-comparative	20 (25.6)
Not available	5 (6.4)
Blinding	
No blinding	46 (59.0)
Single blind	23 (29.5)
Double blind	0 (0)
Not available	9 (11.5)
Control condition	
None	20 (25.6)
Waitlist	16 (20.5)
Standard care	21 (26.9)
Placebo	1 (1.3)
Active	21 (26.9)
Not available	4 (5.1)
Placement in pathway	
Delivered within routine care	43 (55.1)
Delivered outside of routine care	27 (34.6)
Not available	8 (10.3)

**Table 3.** Characteristics of studies relating to number of participants.

Participant characteristics	Values
Enrolled participants	
Number of participants (median)	180
Number of participants (IQR)	80- 380
Number of participants (range)	10- 67,468
Analyzed participants	
Number of participants (median)	129
Number of participants (IQR)	69- 306
Number of participants (range)	10- 948,294

**Table 4.** Characteristics of studies relating to outcomes and results

Characteristics	Studies, n (%)
Dropout at primary endpoint (%)	
0 to 10	26 (33.3)
11 to 20	16 (20.5)
21 to 30	9 (11.5)
31 to 40	4 (5.1)
41 to 50	4 (5.1)
Over 50	7 (9)
Not available	12 (15.4)
Reported information on age	
Yes	63 (80.8)
No	9 (11.5)
Not available	6 (7.7)
Reported information on gender	
Yes	60 (76.9)
No	12 (15.4)
Not available	6 (7.7)
Reported information on ethnicity	
Yes	31 (39.7)
No	40 (51.3)
Not available	6 (7.7)
Identified primary endpoint	
Yes	69 (88.5)
No	4 (5.1)
Not available	5 (6.4)
Met primary endpoint	
Yes	60 (76.9)
No	9 (11.5)
Not specified	4 (5.1)
Not available	5 (6.4)
Included outcome relating to HRQoL, well-being or functioning	
Yes	43 (55.1)
No	29 (37.2)
Not available	6 (7.7)
Included preference-based HRQoL measure	
Yes	15 (19.2)
No	57 (73.1)
Not available	6 (7.7)
Included outcomes relating to resource use	
Yes	21 (19.2)
No	48 (61.5)
Not available	6 (7.7)
Reported approach to adverse events	
Yes	34 (43.6)
No	38 (48.7)
Not available	6 (7.7)

The majority of studies reported conflicts of interest (n=68, 87.2%%) with funding sources from the public sector (n=38, 48.7%), industry (n=20, 25.6%), third sector (n=11, 14.1%), and insurers (n=1, 1.3%). Around half of the studies were registered or had a published protocol (n=37, 47.4%). Half of the studies recruited participants in England (n=43, 55.1%) and most used multi-center designs (n=52, 66.7%). Just over half of the studies were randomized controlled trials (n=42, 53.8%), with the remainder using non-randomized controlled (n=11, 14.1%) and non-comparative (n=20, 25.6%) designs. Half of the studies had no blinding while around a third used single blind designs with allocation status not known to outcome-assessors (n=23, 29.5%). For comparative studies, the control condition was evenly split between waiting list (n=16, 20.5%), standard care (n=21, 26.9%), and active controls (n=21, 26.9%) with one additional study reporting using a placebo control (n=1, 1.3%). Studies using active and placebo controls used a variety of approaches that provided a varying level of therapeutic content. In some cases, standard care and waiting list controls also received therapeutic content as part of their usual care. Study interventions were delivered both as part of (n=43, 55.1%) and outside of routine care (n=27, 34.6%).

For most studies, the primary endpoint was identified or could be determined due to its prominence in reporting (n=60, 76.9%). A total of 54 measures were included as primary endpoints. Half of the studies included an outcome relating to HRQoL, functioning, or well-being (n=43, 55.1%), and 34 different measures were used for these outcomes. A minority of studies included preference-based HRQoL measures that can support the generation of health utilities (n=15, 19.2%) or outcomes relating to impact on resource use (n=21, 26.9%), both of which are valuable for use in economic evaluations. Only a minority of studies reported on adverse events (n=34, 43.6%). Details on all included primary endpoints, and health-related quality of life, functioning, and well-being measures are available in Multimedia Appendix 1.

The median number of enrolled participants was 180 (IQR 300, range=10 to 67,468), with a median of 129 participants analyzed (IQR 237, range 10 to 948,294). The rate of dropout varied from 0% to over 50% of participants, although 12 studies did not report sufficient data to determine dropout from enrolment to analysis. Where the primary endpoint was identified, it was met in the majority of studies (n=60, 76.9%). The majority of studies reported on basic demographic information relating to participant age (n=63, 80.8%) and gender (n=60, 76.9%) but fewer studies reported on participant ethnicity (n=31, 39.7%).

### Supporting Evidence for DMHTs

For five technologies, no relevant supporting evidence was identified during the evaluation. Each of these technologies was recommended for use in research only. 25 technologies had relevant supporting evidence, with most of these supported by one (n=12, 40%) or two (n=4, 13.3%) studies, with a range of between 1 and 21 studies. Ongoing studies were identified for 15 technologies. These were a range of designs, including randomized controlled trials, real-world evidence, and implementation studies.

Most technologies had at least one study that used elements of a high-quality study design (Table 5). Over half of these technologies were supported by studies with randomized methods (n=15, 60%), and this was combined with other elements of high-quality study design, including notably blinding of outcome assessors (n=14, 46.7%%). Most studies also included at least one element of study design that could support NICE decision-making on the use of technologies in the NHS in England. The most common elements were inclusion of outcomes relating to HRQoL, functioning, or well-being (n=19, 76%) and recruitment in England or the rest of the UK (n=18, 60%).

Eight technologies were supported by at least one study with all elements of a high-quality study design and relevance to decision-making for use in the NHS in England. Three technologies were supported by multiple studies that each included all of these elements. Seven technologies included all of these elements but were split across 2 or more different studies.

**Table 5.** Extent of evidence supporting DMHTs^a^ across available studies.

Evidence	Technologies with≥1 supporting studies (n=25), n (%)	Technologies (n=30), n (%)
≥1 study with randomized methods	15 (60.0)	15 (50.0)
≥1 study with blinding	14 (56.0)	14 (46.7)
≥1 study reporting basic demographic information	19 (76.0)	19 (63.3)
≥1 study with defined primary endpoint	22 (88.0)	22 (73.3)
≥1 study with primary endpoint met	19 (76.0)	19 (63.3)
≥1 study including health-related quality of life, functioning, or well-being endpoint	19 (76.0)	19 (63.3)
≥1 study including adverse event reporting	16 (64.0)	16 (53.3)
≥1 study with results specific to England or rest of the United Kingdom	18 (72.0)	18 (60.0)
≥1 study with all of the above	8 (32.0)	8 (26.7)
≥2 studies with all of the above	3 (12.0)	3 (10.0)
All of the above split across ≥2 or more different studies	7 (28.0)	7 (23.3)

a DMHT: digital mental health technologies.

### Content of Evidence Generation Plans

Evidence generation plans were available for seven NICE evaluations for early use. These evidence generation plans outlined seven areas where additional evidence was essential (Table 6). The most common evidence gap that was judged as essential was on costs associated with delivering DMHTs and impact on resource use (n=6). This was followed by further evidence on effectiveness compared to relevant comparators (n=5), adverse events (n=4), appropriate outcomes (n=4), HRQoL (n=2), user experience and engagement (n=1), and quality of data from digital sources (n=1).

**Table 6.** Information from NICE^a^ evidence generation plans on evidence gaps.

NICE^a^ Evaluation	Population	Effectiveness compared to relevant comparators	Appropriate outcomes	Health-related quality of life	Adverse events	User experience and engagement	Cost of delivery and resource use	Subgroups	Quality of information from digital sources
HTE3 [19]	—^b^	E^c^	—	S^d^	—	S	—	S	—
HTE8 [20]	—	E	E	S	E	S	E	—	—
HTE9 [21]	—	E	E	S	E	S	E	—	—
HTE15 [22]	S	E	—	S	E	E	E	—	—
HTE17 [23]	—	E	—	—	E	S	E	—	—
HTE25 [24]	—	—	—	E	—	—	E	S	—
HTE30 [25]	—	—	E	E	—	S	E	—	E

a NICE: National Institute for Health and Care Excellence.

b Indicates the evidence gap was not mentioned.

c E indicates evidence gap is essential for future committee decision-making.

d S indicates evidence gap can support future committee decision-making.

Four areas where additional evidence could support decision-making were also mentioned in evidence generation plans. Two of these were for evidence gaps that were essential for other evaluations, including on user experience and engagement (n=5) and HRQoL (n=4), and two related to additional evidence gaps on effectiveness for specific population subgroups (n=2) and on determining the population that would benefit most from the DMHT (n=1).

Evidence generation plans also provide advice on the most appropriate study designs to support further decision-making. These included robust, well-designed randomized controlled trials and approaches using real-world data with statistical methods that can adequately control for confounding variables (eg, propensity score matching, controlled interrupted time series), which were all deemed to be feasible for these technologies. For some technologies, it was suggested that some evidence gaps could be addressed through additional analysis of already available trial data.

## Discussion

### Principal Findings

In this study, we used a cross-sectional analysis to examine evidence supporting NICE evaluations for DMHTs. This analysis included nine NICE evaluations with recommendations for 30 DMHTs. These DMHTs were supported by a total of 78 studies.

We found that studies evaluating DMHTs often use high-quality methods that provide valuable information on effectiveness and cost-effectiveness. A number of these studies have included elements that are reportedly difficult to incorporate into evaluations of digital health technologies, including randomisation and blinding [28]. Our findings show that there is high-quality evidence available for DMHTs covering various areas of importance and that this evidence accrues across multiple studies, which build to provide a package of evidence to support decision-making. Although there remain key evidence gaps which could be addressed at earlier stages of evidence generation or during early stages of adoption in health services. In some cases, the evidence developed across these studies was sufficient to provide recommendations for routine use in the NHS in England. In other cases, evidence suggested that DMHTs could play a role in health services but there remained uncertainties about their impact and there are a series of common evidence gaps that these DMHTs needed to address prior to further review.

Despite finding supporting high-quality evidence for many DMHTs, we also found that some DMHTs that could help address health system priorities do not appear to have evidence to support claims of their effectiveness and value for money. There needs to be a coordinated focus from stakeholders across the health system, including developers, health professionals, and policymakers, to ensure that DMHTs are supported by appropriate evidence and that they are not adopted into health services until sufficient evidence is available.

### Comparison With Previous Studies

Our findings on study characteristics and common evidence gaps align with previous studies on evidence for DMHTs [3,6,7]. For example, our findings suggest that despite some increases in reporting of adverse events over time, improving monitoring and reporting of adverse events remains a priority [7,10]. This review does provide a more nuanced picture of reporting of adverse events, though, and suggests that developers may focus on recording and reporting adverse events in some but not all of their supporting studies. This approach does not appear to be in line with best practice for health research [29] or with regulatory requirements [30] but shows that valuable information can be found by reviewing the full evidence package for a DMHT. Similarly, evidence on user experience and engagement has previously been cited as a challenge for DMHTs with high levels of discontinuation [11], and our findings suggest that this is a common evidence gap for DMHTs evaluated by NICE.

We also identified additional evidence gaps that have had less discussion in previous research but are important to support decision-making. In order to assess the impact of introducing DMHTs into health services, evidence needs to focus on the benefits compared with currently available care. This was identified as a key gap, as comparative evidence was either not available or studies used comparators that did not reflect standard care in England. Studies did not commonly include preference-based HRQoL measures. These measures can be used to generate health utilities, and their omission causes challenges in assessing cost-effectiveness. This could be addressed by including appropriate measures such as the EuroQol-5 Dimensions (EQ-5D), which is NICE’s preferred instrument for capturing HRQoL, in early evaluations and subsequent trials.

Evidence on the cost of delivering DMHTs and their impact on short- and long-term resource use was the most common evidence gap and was highlighted as an essential area to address. Developers should be clear about licensing and pricing models, and there needs to be an additional focus on developing evidence on the impact on contacts with health professionals, hospitalization, and other relevant outcomes. For some newer DMHTs that are able to collect and analyse large amounts of data, there was also uncertainty about the quality of this data and whether systems were being designed to capture a range of important variables for use within analysis.

Our findings also highlight that challenges that are present in other areas of mental health research are also present for DMHTs. As with previous studies, we found a lack of consistency in the outcomes used for primary endpoints in DMHT studies, as well as a wide range of possible outcomes for measuring HRQoL, functioning, and well-being [9]. Some level of variability is to be expected across different populations and different purposes. However, there was variability in studies for the same or similar DMHTs, which could have implications for assessing comparative effectiveness. Alongside this variability, we also saw a reliance on condition-specific symptom scales and more limited use of other patient-relevant outcome measures, including measures of HRQoL, functioning, and well-being, and other outcomes that matter to patients (eg, relapse, hospitalization, access to services, waiting times). Greater use of these types of outcomes could help ensure that DMHTs align with the goals of users and also address health system priorities.

### Implications for Research and Practice

Our findings have implications for future research and could help guide evidence generation for new and existing DMHTs. We identified a series of common evidence gaps that could help inform study design and could be addressed earlier during the evidence generation process. If these gaps could be addressed earlier in the process, they could increase certainty and allow a wider range of DMHTs to be recommended to support health system priorities. These findings emphasize the need for developers who are aiming for implementation in specific settings to review content from relevant HTA agencies and other decision-makers. To achieve this, there is a wide range of publicly available resources, such as the NICE Evidence Standards Framework [31], and developers may benefit from accessing expertise from advisory services, such as NICE Advice [32]. There are also similar resources that can provide information on using real-world evidence to address uncertainty [33]. We also identified that less than half of the studies supporting DMHTs were registered or had a published protocol, and this was particularly prevalent for nonrandomized studies. The NICE real-world evidence framework encourages developers to publish protocols with pre-specified analyses for studies of comparative effects [33]. This is a further practical step that could be taken to improve the transparency of evidence for DMHTs.

We also identified that there was limited or no evidence to support decision-making on the use of some DMHTs despite them aligning with areas of unmet need for the health system. For some of these DMHTs, this may be because they have been developed more recently and are actively developing evidence but were included in a NICE evaluation alongside DMHTs with more mature evidence. Developers should ensure that they generate sufficient evidence to meet regulatory requirements and to support health technology assessment [8]. This would help support consideration of the potential impact of their technologies and ensure that patients can benefit from the full range of available DMHTs, if they can be demonstrated to be safe, effective, and provide value for money.

### Strengths and Limitations

Our study focuses on a set of NICE evaluations of DMHTs. These evaluations are prioritized by NICE because they represent a priority for the NHS in England, and it has been identified that there are at least some relevant DMHTs with sufficient evidence to make recommendations. This is a key strength of the study because it allows us to examine whether DMHTs that could support national priorities are supported by evidence. It also provides a naturalistic account of evidence available to support decision-making on use at a particular point in time. However, this approach also means there is a substantial level of selection bias in the included DMHTs and our findings may not be reflective of evidence across the broader field of digital mental health. In addition, our approach means that we have a snapshot of evidence for included DMHTs at a specific time point. DMHTs are likely to be generating evidence across their lifecycle, and this study should be revisited to explore how evidence supporting DMHTs changes and whether DMHTs are able to address evidence gaps highlighted in this study.

A further limitation is that we were reliant on evidence that was identified during NICE evaluations and is publicly available. In assessments for early use, a pragmatic approach to identification and selection of evidence is used to allow rapid assessment and to ensure applicability of evidence to the scope of an evaluation. In addition, in several cases, DMHTs were supported by unpublished data which were provided with confidentiality during NICE evaluations but could not be reflected in our findings. For both reasons, this may mean we are not able to capture all available supporting evidence. Our study also focused on evidence of effectiveness and cost-effectiveness, which play a key role in HTA. However, decision-making is also supported by information on a broader set of factors, including the potential for technologies to address unmet needs and the experiences and views of patients and health professionals. These factors could not be captured in this study.

### Conclusions

Digital mental health technologies (DMHTs) are playing an increasing role in mental health services. To achieve their potential, DMHTs need to demonstrate that they are effective and present value for money. However, the quality of evidence for DMHTs is variable and there are concerns that evidence is not sufficient to support decision-making. Our study highlights that some DMHTs are supported by high-quality studies and that evidence to support DMHTs is likely to be developed across multiple studies. There remain key evidence gaps that could be addressed earlier in evidence generation to provide a stronger case for adoption.

Developers should ensure that their evidence generation is aligned with the expectations of decision-makers in relevant settings to reduce uncertainty in these areas. There should also be a greater focus in the field of digital mental health on generating evidence on the cost of delivering DMHTs, their impact on resource use, and on other outcomes that matter to patients, and ensuring that all of these data are compared with current and relevant care options within health services.

## Supplementary material

10.2196/85635 Multimedia Appendix 1 Details of measures included in studies.

## Abbreviations

ADHD: attention deficit hyperactivity disorder
DMHT: digital mental health technologies
HRQoL: health-related quality of life
HTA: Health Technology Assessment
NHS: National Health Service
NICE: National Institute for Health and Care Excellence

## References

[1] Hollis C, Sampson S, Simons L (2018). Identifying research priorities for digital technology in mental health care: results of the James Lind Alliance Priority Setting Partnership. Lancet Psychiatry.

[2] Smith KA, Blease C, Faurholt-Jepsen M (2023). Digital mental health: challenges and next steps. BMJ Ment Health.

[3] Torous J, Bucci S, Bell IH (2021). The growing field of digital psychiatry: current evidence and the future of apps, social media, chatbots, and virtual reality. World Psychiatry.

[4] Greaves F, Joshi I, Campbell M, Roberts S, Patel N, Powell J (2018). What is an appropriate level of evidence for a digital health intervention?. The Lancet.

[5] Görgens M, Cheikh N, Wilkinson T (2021). Digital health interventions: an evidence gap map report (English). https://documents.worldbank.org/en/publication/documents-reports/documentdetail/858961624292229020.

[6] Torous J, Linardon J, Goldberg SB (2025). The evolving field of digital mental health: current evidence and implementation issues for smartphone apps, generative artificial intelligence, and virtual reality. World Psychiatry.

[7] Linardon J, Xie Q, Swords C, Torous J, Sun S, Goldberg SB (2025). Methodological quality in randomised clinical trials of mental health apps: systematic review and longitudinal analysis. BMJ Ment Health.

[8] Hopkin G, Branson R, Campbell P (2024). Considerations for regulation and evaluation of digital mental health technologies. Digit HEALTH.

[9] Juul S, Faltermeier P, Siddiqui F (2025). Challenges in the selection and measurement of outcomes in psychiatric trials. BMJ EBM.

[10] Gómez Bergin AD, Valentine AZ, Rennick-Egglestone S, Slade M, Hollis C, Hall CL (2023). Identifying and categorizing adverse events in trials of digital mental health interventions: narrative scoping review of trials in the international standard randomized controlled trial number registry. JMIR Ment Health.

[11] Smith KA, Ward T, Lambe S (2025). Engagement and attrition in digital mental health: current challenges and potential solutions. NPJ Digit Med.

[12] (2023). What we do. NICE.

[13] (2022). Digital health. NICE.

[14] (2025). NICE healthtech programme manual. NICE.

[15] (2025). Published: guidance, quality standards and advice. NICE.

[16] Hopewell S, Chan AW, Collins GS (2025). CONSORT 2025 statement: updated guideline for reporting randomised trials. BMJ.

[17] Elm E von, Altman DG, Egger M, Pocock SJ, Gøtzsche PC, Vandenbroucke JP (2007). Strengthening the reporting of observational studies in epidemiology (STROBE) statement: guidelines for reporting observational studies. BMJ.

[18] Kumar A, Ross JS, Patel NA, Rathi V, Redberg RF, Dhruva SS (2023). Studies of prescription digital therapeutics often lack rigor and inclusivity.. Health Aff (Millwood).

[19] (2023). NICE. Guided self-help digital cognitive behavioural therapy for children and young people with mild to moderate symptoms of anxiety or low mood: early value assessment (HTE3).

[20] (2023). NICE. Digitally enabled therapies for adults with depression (HTE8).

[21] (2023). NICE. Digitally enabled therapies for adults with anxiety disorders (HTE9).

[22] (2023). NICE. Virtual reality technologies for treating agoraphobia or agoraphobic avoidance: early value assessment (HTE15).

[23] (2024). NICE. Digital health technologies to help manage symptoms of psychosis and prevent relapse in adults and young people: early value assessment (HTE17).

[24] (2025). NICE. Digital therapy for chronic tic disorders and Tourette syndrome: early value assessment (HTE25).

[25] (2025). NICE. Digital front door technologies to gather service user information for NHS Talking Therapies for anxiety and depression assessments: early value assessment (HTE30).

[26] (2024). NICE. Digital technologies for assessing attention deficit hyperactivity disorder (DG60).

[27] (2022). NICE. Sleepio to treat insomnia and insomnia symptoms (MTG70).

[28] Guo C, Ashrafian H, Ghafur S, Fontana G, Gardner C, Prime M (2020). Challenges for the evaluation of digital health solutions-A call for innovative evidence generation approaches. NPJ Digit Med.

[29] (2024). Health research authority. Safety and progress reports (other research) procedural table.

[30] (2024). MHRA. Notify MHRA about a clinical investigation for a medical device.

[31] (2022). NICE. Evidence Standards Framework (ESF) for digital health technologies.

[32] (2025). NICE. NICE Advice service.

[33] NICE (2022). NICE real-world evidence framework. https://www.nice.org.uk/corporate/ecd9.

[34] NICE guidance. NICE.

