# Supplementary material for: Strength of Evidence to Support Decision-Making on the Use of Digital Mental Health Technologies in NICE Evaluations: Cross-Sectional Analysis of Studies
Source: JMIR Ment Health. 2026 Apr 7;13:e85635. doi: 10.2196/85635 (PMC13056029; doi:10.2196/85635)
Supplement: Multimedia Appendix 1 [file mental-v13-e85635-s001.docx]

**Multimedia Appendix 1. Details of measures included in studies**

**Table 1. Measures included as primary endpoints in identified studies**

| **Measure** | **Number of studies** |
| --- | --- |
| Anxiety Disorders Interview Schedule for DSM-5, Child and Parent Versions (ADIS-C/P) | 1 |
| Beck Anxiety Inventory (BAI) | 2 |
| Beck Depression Inventory (BDI) | 1 |
| Beck Depression Inventory-II (BDI-II) | 6 |
| Beliefs About Voices Questionnaire- Revised (BAVQ-R) | 1 |
| British Columbia Cognitive Complaints Inventory (BC-CCI) | 1 |
| Change in 24 hour ambulatory systolic blood pressure | 1 |
| Change in phobia behaviours | 1 |
| Child Anxiety Impact Scale- Parent (CAIS-P) | 1 |
| Children's Depression Scale (CDS) | 1 |
| Clinical Outcomes in Routine Evaluation – Outcome Measure (CORE-OM) | 4 |
| Clinician-Administered PTSD Scale for DSM-5 (CAPS-5) | 1 |
| Cost of healthcare resource use | 1 |
| Depression Anxiety Stress Scale-21 (DASS-21) | 1 |
| Empowerment Rating Scale (ERS) | 1 |
| General Anxiety Disorder (GAD-7) | 15 |
| Geriatric Anxiety Inventory (GAI) | 1 |
| Geriatric Depression Scale (GDS) | 1 |
| Glasgow Sleep Impact Index (GSII) | 1 |
| Green et al Paranoid Thoughts Scale (GPTS) | 2 |
| Initiation of ADHD medication | 1 |
| Initiation of sleep medication | 1 |
| Insomnia Severity Index (ISI) | 5 |
| Liebowitz Social Anxiety Scale (LSAS) | 2 |
| Montgomery Asberg-Depression Rating Scale, Short Version (MADRS) | 1 |
| Number of appointments to confirm or exclude ADHD diagnosis | 5 |
| Number of days from referral to assessment to diagnostic decision on ADHD | 1 |
| Number of self-referrals to psychological therapy services | 1 |
| Numbers of days from first appointment to reach diagnostic decision on ADHD | 2 |
| Oxford Agoraphobic Avoidance Scale (O-AS) | 1 |
| Patient Health Questionnaire (PHQ-9) | 17 |
| Patient Satisfaction Questionnaire Short Form (PSQSF) | 1 |
| Patient-Reported Outcomes Measurement Information System (PROMIS) | 1 |
| Positive and Negative Syndrome Scale (PANSS) Positive Symptoms Subscale | 1 |
| Presence of ADHD diagnosis | 1 |
| Proportion of time in bed asleep (sleep efficiency) | 1 |
| Psychotic Symptom Rating Scales (PSYRATS) - Total | 1 |
| Psychotic Symptom Rating Scales, Auditory Hallucinations subscale (PSYRATS-AH) | 1 |
| Psychotic Symptom Rating Scales, Auditory Hallucinations subscale (PSYRATS-AH), Distress dimension | 1 |
| PTSD Checklist for DSM-5 (PCL-5) | 1 |
| PTSD Symptom Scale - Interview (PSS-I) | 1 |
| Quick Inventory of Depressive Symptomatology-Self-Report (QIDS-SR) | 2 |
| Revised Children's Anxiety and Depression Scale- Parent (RCADS-P) | 2 |
| Short Form Health Survey 12-item, Mental Component Score (SF-12 MCS) | 2 |
| Sleep Condition Indicator (SCI) | 3 |
| Sleep Condition Indicator, Short Form (SCI-02) | 1 |
| Social Anxiety Disorder Composite | 1 |
| Specific Psychotic Experiences Questionnaire (SPEQ) Hallucinations | 1 |
| Spence Children's Anxiety Scale - Parent - Brief (SCAS-P-8) | 1 |
| Treatment Acceptability Questionnaire (TAQ) | 1 |
| Warwick-Edinburgh Mental Wellbeing Scale (WEMWBS) | 1 |
| Work and Social Adjustment Scale (WSAS) | 7 |
| Yale Global Tic Severity Scale- Total Tic Severity Score (YGTSS-TTSS) | 2 |
| Yale-Brown Obsessive-Compulsive Scale Modified for Body Dysmorphic Disorder (BDD-YBOCS) | 2 |

**Table 2. Measures included as primary endpoints in identified studies by NICE evaluation**

| **NICE Evaluation** | **Measure** | **Number of studies** |
| --- | --- | --- |
| HTE3 | Anxiety Disorders Interview Schedule for DSM-5, Child and Parent Versions (ADIS-C/P) | 1 |
|  | Child Anxiety Impact Scale- Parent (CAIS-P) | 1 |
|  | Liebowitz Social Anxiety Scale (LSAS) | 1 |
|  | Revised Children's Anxiety and Depression Scale- Parent (RCADS-P) | 2 |
|  | Spence Children's Anxiety Scale - Parent - Brief (SCAS-P-8) | 1 |
| HTE8 | Beck Anxiety Inventory (BAI) | 1 |
|  | Beck Depression Inventory (BDI) | 1 |
|  | Beck Depression Inventory-II (BDI-II) | 5 |
|  | Clinical Outcomes in Routine Evaluation – Outcome Measure (CORE-OM) | 3 |
|  | General Anxiety Disorder (GAD-7) | 5 |
|  | Geriatric Anxiety Inventory (GAI) | 1 |
|  | Geriatric Depression Scale (GDS) | 1 |
|  | Montgomery Asberg-Depression Rating Scale, Short Version (MADRS) | 1 |
|  | Patient Health Questionnaire (PHQ-9) | 9 |
|  | Quick Inventory of Depressive Symptomatology-Self-Report (QIDS-SR) | 1 |
|  | Short Form Health Survey 12-item, Mental Component Score (SF-12 MCS) | 1 |
|  | Cost of healthcare resource use | 1 |
|  | Work and Social Adjustment Scale (WSAS) | 3 |
| HTE9 | Beck Anxiety Inventory (BAI) | 1 |
|  | Beck Depression Inventory-II (BDI-II) | 1 |
|  | Clinical Outcomes in Routine Evaluation – Outcome Measure (CORE-OM) | 1 |
|  | Clinician-Administered PTSD Scale for DSM-5 (CAPS-5) | 1 |
|  | Depression Anxiety Stress Scale-21 (DASS-21) | 1 |
|  | General anxiety disorder (GAD-7) | 8 |
|  | Liebowitz Social Anxiety Scale (LSAS) | 1 |
|  | Patient Health Questionnaire (PHQ-9) | 6 |
|  | PTSD Checklist for DSM-5 (PCL-5) | 1 |
|  | PTSD Symptom Scale - Interview (PSS-I) | 1 |
|  | Short Form Health Survey 12-item, Mental Component Score (SF-12 MCS) | 1 |
|  | Social Anxiety Disorder Composite | 1 |
|  | Work and Social Adjustment Scale (WSAS) | 3 |
|  | Yale-Brown Obsessive-Compulsive Scale Modified for Body Dysmorphic Disorder (BDD-YBOCS) | 2 |
| HTE15 | Oxford Agoraphobic Avoidance Scale (O-AS) | 1 |
|  | Change in phobia behaviours | 1 |
| HTE17 | Beliefs About Voices Questionnaire- Revised (BAVQ-R) | 1 |
|  | Children's Depression Scale (CDS) | 1 |
|  | Empowerment Rating Scale (ERS) | 1 |
|  | Green et al Paranoid Thoughts Scale (GPTS) | 1 |
|  | Positive and Negative Syndrome Scale (PANSS) Positive Symptoms Subscale | 1 |
|  | Psychotic Symptom Rating Scales (PSYRATS) - Total | 1 |
|  | Psychotic Symptom Rating Scales, Auditory Hallucinations subscale (PSYRATS-AH) | 1 |
|  | Psychotic Symptom Rating Scales, Auditory Hallucinations subscale (PSYRATS-AH), Distress dimension | 1 |
|  | Relapse | 1 |
| HTE25 | Yale Global Tic Severity Scale- Total Tic Severity Score (YGTSS-TTSS) | 2 |
| HTE30 | General anxiety disorder (GAD-7) | 1 |
|  | Number of self-referrals to psychological therapy services | 1 |
|  | Patient Health Questionnaire (PHQ-9) | 1 |
| MTG70 | British Columbia Cognitive Complaints Inventory (BC-CCI) | 1 |
|  | General anxiety disorder (GAD-7) | 1 |
|  | Glasgow Sleep Impact Index (GSII) | 1 |
|  | Green et al Paranoid Thoughts Scale (GPTS) | 1 |
|  | Insomnia Severity Index (ISI) | 5 |
|  | Change in 24 hour ambulatory systolic blood pressure | 1 |
|  | Patient Health Questionnaire (PHQ-9) | 1 |
|  | Patient Satisfaction Questionnaire Short Form (PSQSF) | 1 |
|  | Patient-Reported Outcomes Measurement Information System (PROMIS) | 1 |
|  | Quick Inventory of Depressive Symptomatology-Self-Report (QIDS-SR) | 1 |
|  | Sleep Condition Indicator (SCI) | 3 |
|  | Sleep Condition Indicator, Short Form (SCI-02) | 1 |
|  | Proportion of time in bed asleep (sleep efficiency) | 1 |
|  | Initiation of sleep medication | 1 |
|  | Specific Psychotic Experiences Questionnaire (SPEQ) Hallucinations | 1 |
|  | Treatment Acceptability Questionnaire (TAQ) | 1 |
|  | Warwick-Edinburgh Mental Wellbeing Scale (WEMWBS) | 1 |
|  | Work and Social Adjustment Scale (WSAS) | 1 |
| DG60 | Initation of ADHD medication | 1 |
|  | Number of appointments to confirm or exclude ADHD diagnosis | 5 |
|  | Number of days from referral to assessment to diagnostic decision on ADHD | 1 |
|  | Numbers of days from first appointment to reach diagnostic decision on ADHD | 2 |
|  | Presence of ADHD diagnosis at 1 year | 1 |

**Table 3. Measures relating to health-related quality of life, well-being and functioning included in identified studies**

| Measure | Number of studies |
| --- | --- |
| 1-item on concentration in class | 1 |
| 2-items from Work Productivity and Impairment Questionnaire (WPIQ) | 1 |
| 2-items on life satisfaction and work productivity | 1 |
| 5-items from Index of Job Satisfaction | 1 |
| 6-items on daytime functioning | 1 |
| Child Anxiety Impact Scale, Parent (CAIS-P) | 2 |
| Child Anxiety Life Interference Scale (CALIS) | 1 |
| Child Health Utility 9- dimension (CHU-9D), parent * | 1 |
| Child Health Utility 9- dimension (CHU-9D), self * | 1 |
| Child Outcome Rating Scale (CORS) | 1 |
| Children’s Assessment of Participation and Enjoyment (CAPE) | 1 |
| Clinical Outcomes in Routine Evaluation – Outcome Measure (CORE-OM) | 8 |
| Employment Self Report Questionnaire | 2 |
| EuroQol- 5 Dimension- 3 Level (EQ-5D-3L) * | 3 |
| EuroQol- 5 Dimension- 5 Level (EQ-5D-5L) * | 5 |
| EuroQol- 5 Dimension- Youth (EQ-5D-Y) * | 1 |
| Gilles de la Tourette Syndrome-Quality of Life Scale (C&A-GTS-QoL) | 2 |
| Glasgow Sleep Impact Index (GSII) | 1 |
| KIDSCREEN, parent | 1 |
| KIDSCREEN, self | 1 |
| Manchester Short Assessment of Quality of Life (MANSA) | 2 |
| Patient-Reported Outcomes Measurement Information System (PROMIS) * | 3 |
| Process of Recovery Questionnaire (QPR) | 1 |
| Quality of Life Enjoyment and Satisfaction Questionnaire Short Form (Q-LES-Q-SF) | 2 |
| Recovering Quality of Life (ReQoL) * | 1 |
| Sheehan Disability Scale (SDS) | 3 |
| Short Form Health Survey 12-item (SF-12) * | 6 |
| Short Form Health Survey 36-item (SF-36) * | 1 |
| Social Participation Scale (SPS) | 1 |
| Social Satisfaction Scale (SCS) | 1 |
| Warwick-Edinburgh Mental Wellbeing Scale (WEMWBS) | 2 |
| Work and Productivity and Impairment Questionnaire (WPIQ) | 1 |
| Work and Social Adjustment Scale (WSAS) | 16 |
| World Health Organization’s Quality of Life Questionnaire (WHOQOL-BREF) | 2 |

* indicates measures with associated value set

**Table 4. Measures relating to health-related quality of life, well-being and functioning included in identified studies by NICE evaluation**

| **NICE Evaluation** | **Measure** | **Number of studies** |
| --- | --- | --- |
| HTE3 | 1-item on concentration in class | 1 |
|  | Child Anxiety Impact Scale, Parent (CAIS-P) | 2 |
|  | Child Anxiety Life Interference Scale (CALIS) | 1 |
|  | Child Outcome Rating Scale (CORS) | 1 |
|  | Social Participation Scale (SPS) | 1 |
|  | Social Satisfaction Scale (SCS) | 1 |
| HTE8 | Clinical Outcomes in Routine Evaluation – Outcome Measure (CORE-OM) | 6 |
|  | Employment Self Report Questionnaire | 1 |
|  | EuroQol- 5 Dimension- 3 Level (EQ-5D-3L) * | 2 |
|  | EuroQol- 5 Dimension- 5 Level (EQ-5D-5L) * | 1 |
|  | Patient-Reported Outcomes Measurement Information System (PROMIS) * | 1 |
|  | Sheehan Disability Scale (SDS) | 1 |
|  | Short Form Health Survey 12-item (SF-12) * | 5 |
|  | Short Form Health Survey 36-item (SF-36) * | 1 |
|  | Work and Social Adjustment Scale (WSAS) | 7 |
|  | World Health Organization’s Quality of Life Questionnaire (WHOQOL-BREF) | 2 |
| HTE9 | Clinical Outcomes in Routine Evaluation – Outcome Measure (CORE-OM) | 2 |
|  | Employment Self Report Questionnaire | 1 |
|  | EuroQol- 5 Dimension- 3 Level (EQ-5D-5L) * | 1 |
|  | Patient-Reported Outcomes Measurement Information System (PROMIS) * | 1 |
|  | Quality of Life Enjoyment and Satisfaction Questionnaire Short Form (Q-LES-Q-SF) | 2 |
|  | Sheehan Disability Scale (SDS) | 2 |
|  | Short Form Health Survey 12-item (SF-12) * | 1 |
|  | Work and Social Adjustment Scale (WSAS) | 7 |
| HTE15 | Children’s Assessment of Participation and Enjoyment (CAPE) | 1 |
|  | EuroQol- 5 Dimension- 5 Level (EQ-5D-5L) * | 1 |
|  | Process of Recovery Questionnaire (QPR) | 1 |
|  | Recovering Quality of Life (ReQoL) * | 1 |
| HTE17 | EuroQol- 5 Dimension- 3 Level (EQ-5D-3L) * | 1 |
|  | EuroQol- 5 Dimension- 5 Level (EQ-5D-5L) * | 2 |
|  | Manchester Short Assessment of Quality of Life (MANSA) | 2 |
| HTE25 | Child Health Utility 9- dimension (CHU-9D), parent * | 1 |
|  | Child Health Utility 9- dimension (CHU-9D), self * | 1 |
|  | Gilles de la Tourette Syndrome-Quality of Life Scale (C&A-GTS-QoL) | 2 |
|  | KIDSCREEN, parent | 1 |
|  | KIDSCREEN, self | 1 |
| MTG70 | 2-items from Work Productivity and Impairement Questionnaire (WPIQ) | 1 |
|  | 2-items on life satisfaction and work productivity | 1 |
|  | 5-items from Index of Job Satisfaction | 1 |
|  | 6-items on daytime functioning | 1 |
|  | Glasgow Sleep Impact Index (GSII) | 1 |
|  | Patient-Reported Outcomes Measurement Information System (PROMIS) * | 1 |
|  | Warwick-Edinburgh Mental Wellbeing Scale (WEMWBS) | 2 |
|  | Work and Productivity and Impairment Questionnaire (WPIQ) | 1 |
|  | Work and Social Adjustment Scale (WSAS) | 2 |
| DG60 | EuroQol- 5 Dimension- Youth (EQ-5D-Y) * | 1 |

* indicates measures with associated value set
